# 539. Determining the Value of Screening and Decolonization in Burn Unit Management

**DOI:** 10.1093/jbcr/irag033.236

**Published:** 2026-04-06

**Authors:** Chinaemelum C Akpunonu, Jemima Carroll, Michael R Young, Nidhi D Aravapalli, Shandra Day, Ariel Rodgers, John H Loftus, Nicole P Bernal

**Affiliations:** The Ohio State University Wexner Medical Center, Columbus, OH; The Ohio State University Wexner Medical Center, Columbus, OH; The Ohio State Comprehensive Burn Center, Columbus, OH; The Ohio State Comprehensive Burn Center, Columbus, OH; The Ohio State University Wexner Medical Center, Columbus, OH; The Ohio State Comprehensive Burn Center, Columbus, OH; The Ohio State Comprehensive Burn Center, Columbus, OH; Ohio State University Comprehensive Burn Center, Columbus, OH

## Abstract

**Introduction:**

Burn wound infection is a major complication that can lead to longer length of stays (LOS), graft loss, additional procedures, unplanned readmissions, and sepsis. Methicillin-resistant *Staphylococcus aureus* (MRSA) has been reported as one of the more serious wound infections among burn patients. Previous studies have shown that hospitals with MRSA screening and decolonization protocols have significantly lower incidences of MRSA during hospitalizations, decreases in CAUTI and CLABSIs, and shorter LOS. Our institution does not routinely screen or isolate for MRSA colonization or wound infections. A concern for increased MRSA infections in multiple burn patients led to the development of a quality improvement (QI) project with our Infectious Disease team to screen for MRSA, decolonize positive patients, and track hospital outcomes and infection rates.

**Methods:**

This project followed new patients who were admitted to the burn service during March 2025. Upon admission, all burn patients were screened for MRSA (axilla and nares) by PCR. Only MRSA+ patients were decolonized with mupirocin in the nares for 5 days and had daily Chlorhexidine Gluconate (CHG) baths with scrubbing under the nails. The MSSA+ and negative patients had wound care that followed our institutional protocol. A patient with a concern for wound infection had cultures taken and appropriate medical and surgical interventions were completed as indicated.

**Results:**

Forty-five burn patients were admitted during the study period. Six were excluded (4 not on burn service, 2 were readmissions). There was a 93% compliance with admission screening (n = 36). Among those screened, 19 (49%) were negative, 10 (26%) were MSSA+, and 7(18%) were MRSA+. All MRSA+ patients completed decolonization. Among the 45 patients, only 2 (4%) developed a wound infection that had MRSA that required treatment. One patient screened positive for MRSA upon readmission, while the other initially screened positive for MSSA and developed MRSA in the wound during the admission. There were no differences in length of stay, or mortality between any of the cohorts.

**Conclusions:**

This small sample size study from a single institution did not demonstrate a benefit for screening and decolonization. Our MRSA infection rate is low. The MSSA and MRSA+ patients had similar outcomes. Based on the initial findings, we are working with our Epidemiology and Infection Prevention team to screen patients without decolonization to determine if colonization leads to wound infections.

**Applicability of Research to Practice:**

Screening and decolonization are an additional expense to an institution that should be done with compelling evidence in infection reduction. If screening and decolonization do not have an impact on infection rates, then this common practice may need to be re-evaluated on a larger scale.

**Funding for the study:**

N/A.

